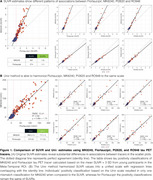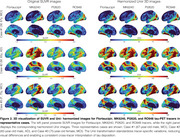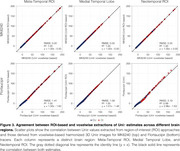# Harmonization of Flortaucipir, MK6240, PI2620 and RO948 with the Uniτ scale

**DOI:** 10.1002/alz70862_110041

**Published:** 2025-12-23

**Authors:** Guilherme Povala, Guilherme Bauer‐Negrini, Bruna Bellaver, Livia Amaral, Firoza Z Lussier, Pamela C.L. Ferreira, Dana L Tudorascu, Quentin Finn, Joseph C. Masdeu, David N. soleimani‐Meigooni, Juan Fortea, Val J Lowe, Hwamee Oh, Brian A. Gordon, Belen Pascual, Pedro Rosa‐Neto, Suzanne L. Baker, Tharick A Pascoal

**Affiliations:** ^1^ University of Pittsburgh, Pittsburgh, PA USA; ^2^ Houston Methodist Research Institute, Houston, TX USA; ^3^ University of California, San Francisco, San Francisco, CA USA; ^4^ Sant Pau Memory Unit, Hospital de la Santa Creu i Sant Pau, Biomedical Research Institute Sant Pau, Barcelona Spain; ^5^ Mayo Clinic, Rochester, MN USA; ^6^ Brown University, Providence, RI USA; ^7^ Washington University in St. Louis, St. Louis, MO USA; ^8^ McGill University, Montreal, QC Canada; ^9^ Lawrence Berkeley National Laboratory, Berkeley, CA USA

## Abstract

**Background:**

Precise cross‐tracer harmonization of tau PET imaging is essential for comparing tau burden across different tracers and studies. Here, we evaluated the performance of the universal tau PET scale (Uniτ) in harmonizing Flortaucipir, MK6240, PI2620, and RO948 tau PET images into a universal scale.

**Method:**

We assessed 485 individuals across aging and AD spectrum scanned head‐to‐head with Flortaucipir and MK6240, and a subset of 90 individuals with additional PI2620 and RO948. We generated tau PET SUVRs using the inferior cerebellar gray matter as reference with a common 8mm FWHM. We estimated Uniτ parameters on a training set (*n* = 200) by fitting a smoothed hyperbolic tangent equation to the Meta‐Temporal ROI anchored in the mean SUVR of young participants and the 90th percentile from cognitively impaired individuals. We compared tau positivity on the Uniτ scale with classifications from SUVRs (mean + 3 SD from Youngs). We applied the same equation to all brain voxels to generate Uniτ 3D images. Then, we extracted mean Uniτ values for key ROIs and correlated them with ROI‐based Uniτ values to evaluate voxel‐wise estimates.

**Result:**

Using tracer‐specific parameters, Uniτ harmonized Flortaucipir, MK6240, PI2620, and RO948 to the same scale, aligning values near the identity line and confirming its applicability across tau PET tracers (Figure 1). For Flortaucipir, Uniτ tau positivity matched original SUVR classifications (ground truth), with only one mismatch for MK6240 (Figure 1). Applying the Uniτ transformation to all brain voxels effectively harmonized 3D images to a common scale, reducing visual variabilities (Figure 2). Furthermore, ROI‐based estimates and those extracted from the 3D Uniτ images were identical (Figure 3).

**Conclusion:**

Our results indicate that tau PET tracers can be harmonized to a common scale using a large head‐to‐head dataset. The Uniτ scale can harmonize entire 3D tau PET images, suggesting that there is no need to use pre‐established ROIs, which would constrain the analysis to a few brain regions. Uniτ is freely accessible across platforms (www.unitau.app) for ROI and 3D tau PET harmonization.